# S100A11 is involved in the progression of colorectal cancer through the desmosome-catenin-TCF signaling pathway

**DOI:** 10.1007/s11626-024-00930-2

**Published:** 2024-06-06

**Authors:** Jin Zhou, Hitoshi Murata, Nahoko Tomonobu, Naoko Mizuta, Atsuko Yamakawa, Ken-ichi Yamamoto, Rie Kinoshita, Masakiyo Sakaguchi

**Affiliations:** 1 Department of Cell Biology, Okayama University Graduate School of Medicine, Dentistry and Pharmaceutical Sciences, 2-5-1 Shikata-Cho, Kita-Ku, Okayama, 700-8558 Japan; 2 Medical Oncology Department of Gastrointestinal Tumors, Liaoning Cancer Hospital & Institute, Cancer Hospital of the Dalian University of Technology, Shenyang, Liaoning, China

**Keywords:** S100A11, Desmosome, TCF signaling, Colorectal cancer

## Abstract

Compiling evidence has indicated that S100A11 expression at high levels is closely associated with various cancer species. Consistent with the results reported elsewhere, we have also revealed that S100A11 is highly expressed in squamous cell carcinoma, mesothelioma, and pancreatic cancers and plays a crucial role in cancer progression when secreted into extracellular fluid. Those studies are all focused on the extracellular role of S100A11. However, most of S100A11 is still present within cancer cells, although the intracellular role of S100A11 in cancer cells has not been fully elucidated. Thus, we aimed to investigate S100A11 functions within cancer cells, primarily focusing on colorectal cancer cells, whose S100A11 is abundantly present in cells and still poorly studied cancer for the protein. Our efforts revealed that overexpression of S100A11 promotes proliferation and migration, and downregulation inversely dampens those cancer behaviors. To clarify how intracellular S100A11 aids cancer cell activation, we tried to identify S100A11 binding proteins, resulting in novel binding partners in the inner membrane, many of which are desmosome proteins. Our molecular approach defined that S100A11 regulates the expression level of DSG1, a component protein of desmosome, by which S100A11 activates the TCF pathway via promoting nuclear translocation of γ-catenin from the desmosome. The identified new pathway greatly helps to comprehend S100A11’s nature in colorectal cancers and others.

**Supplementary Information:**

The online version contains supplementary material available at 10.1007/s11626-024-00930-2.

## Introduction

The S100 proteins are a low-molecular-weight protein of 9–14 kDa and have a Ca^2+^ binding EF-hands motif in their structure. Twenty types of families (S100A1-16, B, G, P, and Z) have been discovered in humans, and they function not only intracellularly but also by being secreted extracellularly (Singh and Ali 2022). S100A11 is one of the S100 proteins aliased as S100C or Calgizzarin. S100A11 is expressed in various tissues, but the expression level differs depending on the tissue (Zhang *et al*. 2021). It has been reported that the S100 family proteins are involved in cancer growth and metastasis (Bresnick *et al*. 2015), and there is a difference in the expression of the S100 proteins between normal tissue and cancer tissue. S100A11 has been reported to be upregulated in various cancers, including pancreatic and colorectal (Meding *et al*. 2012; Zhang *et al*. 2019; Wang *et al*. 2021; Zeng *et al*. 2022; Zheng *et al*. 2023).

We previously reported that S100A11 secreted from pancreatic cancer cells promotes the proliferation of surrounding fibroblasts and is involved in cancer progression (Takamatsu *et al*. 2019). On the other hand, most of S100A11 is still present within cancer cells; however, the intracellular role of S100A11 in cancer cells has not been fully elucidated. Therefore, in this study, we aimed to clarify the unidentified role of S100A11 within cancer cells, typically focused on colorectal cancers, since S100A11 shows relatively high expression in this type of cancer (Saho *et al*. 2016). We herein first report that desmoplakin, desmoglein-1 (DSG1), plakoglobin (γ-catenin), and corneodesmosin, which are proteins involved in cell adhesion, are newly identified as S100A11-interacting proteins. These proteins are the constituent proteins of the desmosome (Muller *et al*. 2021). Desmosomes are localized patchy structures that hold two cells together and are one of the intercellular junctions. Adjacent cells are bound by DSG1 and desmocolin (Cadherins) binding in the extracellular domain. In the intracellular region, γ-catenin and plakophilin act as mediators to anchor the desmosome structure and bind to intermediate filaments. γ-catenin is also involved in adherens junctions (Lessey *et al*. 2022).

Catenin, a protein involved in adherens junctions, includes p120-catenin, α-catenin, β-catenin, and γ-catenin. As lining molecules of cadherins, responsible for cell–cell junctions, they play an essential role in connecting adhered cell membranes to F-actin, one of the cytoskeletons. Among these, β-catenin is involved in adherens junctions and is widely known as a regulator of Wnt/β-catenin signaling. β-Catenin regulates the expression of target genes involved in cell proliferation and survival via the TCF promoter, and β-catenin dysregulation is often associated with colorectal cancer progression (Lessey *et al*. 2022; He and Gan 2023). γ-Catenin has a structural feature with a central armadillo sequence similar to β-catenin. It has also been reported that γ-catenin complements the function of β-catenin and regulates TCF target genes (Maeda *et al*. 2004). Based on these reports, we hypothesized that S100A11 may regulate TCF promoter activity via interaction with γ-catenin and desmosome proteins, which play a role in colorectal cancer outgrowth.

## Materials and methods

### Cell lines

American Type Culture Collection (ATCC; Rockville, MD) was the purchased source for the following cell lines: DLD-1, human colorectal adenocarcinoma cell line; HT-29, human colorectal adenocarcinoma cell line; A-431, human skin epidermoid carcinoma cell line; Hep G2, human hepatocellular carcinoma cell line; Saos-2, human osteosarcoma cell line; PC-3, human prostate adenocarcinoma cell line; T24, human urinary bladder carcinoma cell line; Caki-1, human kidney carcinoma clear cell line; and HeLa, human cervix adenocarcinoma cell line. SH-SY5Y, human neuroblastoma cell line, was purchased from the European Collection of Authenticated Cell Cultures (ECACC; Porton Down, Salisbury, UK). PK-8, human prostate adenocarcinoma cell line, and HEK293, human kidney embryonic cell line, were purchased from RIKEN BioResource Center (Tsukuba, Japan). HCT 116 (p21^WAF1/CIP1+/+^), human colorectal adenocarcinoma cell line, was kindly gifted by Dr. Vogelstein (Waldman *et al*. 1995).

### Cell line authentication

DLD-1 cell line was authenticated by the STR analysis (BEX Co., Ltd.) (Fig. S1A). The STR profiles of DLD-1 that we used were judged as compatible with the reference profiles of DLD-1 (CVCL_0248) since the evaluation value (EV) between DLD-1 and DLD-1 (CVCL_0248) was 0.97. Therefore, two cell lines were considered identical. In addition, all the experiments in this study were performed with mycoplasma-free cells, which we confirmed by detecting no appreciable signal of mycoplasma within the Hoechst 33,342 stained cells (Fig. S1*B*).

### Antibodies

The following antibodies were used: rabbit anti-S100A11 (Proteintech, cat# 10,237–1-AP, dilution 1:1000 in Can Get Signal Solution 1 [Toyobo, Osaka, Japan]), rabbit anti-α-Tubulin (Proteintech, Rosemont, IL, cat# 66,031–1-lg, dilution 1:1000 in 10% skim milk), mouse anti-DSG1 (Santa Cruz Biotechnology, cat# sc-137164, dilution 1:10,000 in 10% skim milk), mouse anti-Desmoplakin (Santa Cruz Biotechnology, Dallas, TX, cat# sc-390975, dilution 1:500 in Can Get Signal Solution 1), rabbit anti-β-catenin (Cell Signaling Technology [CST], Danvers, MA, cat# 9582, dilution 1:1000 in Can Get Signal Solution 1), rabbit anti-γ-catenin (CST, cat# 75,550, dilution 1:1000 in 10% skim milk), rabbit anti-cyclin D1 (CST, cat# 55,506, dilution 1:500 in Can Get Signal Solution 1), rabbit anti-c-Myc (CST, cat# 18,583, dilution 1:500 in Can Get Signal Solution 1), rabbit anti-TCF1/TCF7 (CST, cat# 2203, dilution 1:500 in Can Get Signal Solution 1), mouse anti-Myc-Tag (CST, cat# 2276, dilution 1:1000 in 10% skim milk), mouse anti-β-Actin (Sigma-Aldrich, St. Louis, MO, cat # A2228, dilution 1:20,000 in 10% skim milk), and rabbit anti-Histone H3 (Abcam, Cambridge, UK, cat# ab5103, dilution 1:500 in Can Get Signal Solution 1).

### Plasmid constructs

Conventional molecular biological techniques were used to generate the following expression constructs: N-terminal FLAG-tagged human S100A11, C-terminal Myc-tagged human β-catenin, and γ-catenin. These constructs were ligated with the pIDT-SMART (C-TSC) vector (pCMViR-TSC) (Sakaguchi *et al*. 2014). All expression constructs were sequenced to ensure that the fusion was in the correct reading frame and there were no additional mutations.

### Cell transfection and luciferase reporter assay

For plasmid-DNA transfection, DLD-1 cells were transfected with the pCMViR-TSC plasmids carrying several cDNAs of our interest using FuGENE-HD (Promega Biosciences, Madison, WI). The transfection was carried out at 40% cell density. For 12-well plates in transfection experiments, 2 μg of each plasmid construct and its corresponding 4 μl of FuGENE-HD were mixed and used.

For RNA interference, cells were transfected with the indicated siRNA using Lipofectamine RNAiMAX Transfection Reagent (Thermo Fisher Scientific, Waltham, MA). The transfection was carried out at 40% cell density. For 12-well plates in transfection experiments, 2 μl of siRNA (final concentration of 20 nM) and its corresponding 4 μl of Lipofectamine RNAiMAX Transfection Reagent were mixed and used. In this RNA interference, Stealth RNAi siRNA targeting S100A11 (HSS109441 and HSS184493) and Med GC, a Stealth RNAi siRNA Negative Control were purchased from Thermo Fisher Scientific, and those were applied in this study.

For luciferase reporter assay, cells were transfected with the indicated plasmids, which indicated siRNA, a plasmid encoding GFP, and pGL4.49[luc2P/TCF-LEF RE/Hygro] vector (Promega Biosciences). The luminescence intensity of cells incubated with Britelite Plus Reporter Gene Assay System (PerkinElmer, Waltham, MA) was detected by Fluoroskan Ascent FL (Thermo Fisher Scientific). The luciferase activity was normalized to co-transfected GFP fluorescence.

### Immunoprecipitation and LC–MS/MS analysis

For immunoprecipitation, cells were lysed in an ice-cold lysis buffer (40 mM Tris–HCl, pH 7.4, 150 mM NaCl, and 1% Triton X-100). After centrifugation, supernatants were incubated with 20 μl of a 50% slurry of monoclonal anti-FLAG-M2 affinity gel (Merck, Rahway, NJ) for 3 h. Immunoprecipitates were washed three times with the lysis buffer, eluted with 0.1 M Gly-HCl (pH 2.5) solution, and immediately neutralized with 1 M Tris–HCl, pH 9.0. The eluted solution was incubated with trypsin and analyzed by 1100LC/MSD TRAP XCT Ultra (Agilent Technologies, St. Clara, CA).

### Cell fractionation and Western blot analysis

Cell fractionation was done using LysoPure Nuclear and Cytoplasmic Extractor Kit (FUJIFILM Wako Chemicals, Richmond, VA) according to the manufacturer’s instructions. The fractionation of two parts was confirmed by each fraction marker, α-Tubulin and Histone H3.

Western blot analysis was performed under conventional conditions after lysing cells using an SDS sample buffer with PhosphoSTOP (Roche, Basel, Switzerland). Each 5 µg of protein extract was separated by SDS–polyacrylamide gel electrophoresis and electro-transferred onto an Immobilon membrane (Millipore). To detect immunoreactive proteins, we used HRP-conjugated anti-mouse or anti-rabbit secondary antibodies (CST) and Pierce Western Blotting Substrate Plus (Thermo Fisher Scientific).

### Immunohistochemistry

A human colon carcinoma tissue array (US Biomax, Inc) was deparaffinized, and antigen retrieval was performed by conventional microwave treatment using a citric acid solution. The slides were then incubated with anti-S100A11 antibody (Proteintech, Rosemont, IL) and followed Alexa Fluor 594 goat anti-rabbit IgG antibody (Thermo Fisher Scientific) and SYBR Green I Nucleic Acid Gel stain (Thermo Fisher Scientific). The specimens were observed using a confocal laser-scanning microscope (model LSM510; Car: Zeiss Oberkochen, Germany).

### Immunocytochemistry

Cells were fixed with 4% paraformaldehyde for 30 min. After being washed with PBS, the cells were permeabilized with 0.1% TritonX-100 in PBS (PBST) for 20 min, then were incubated with a blocking buffer (10% skim milk in PBST) for 20 min. Next, the cells were incubated with a mouse anti-FLAG M2 monoclonal antibody (Sigma-Aldrich, cat# F1804, dilution 1:100 in the blocking buffer) and a rabbit anti-DSG1 antibody (Proteintech, cat# 24,857–1-AP, dilution 1:100 in the blocking buffer) for 18 h at 4°C. After being washed in PBST, the cells were incubated with Alexa 594 goat anti-mouse IgG antibody (Thermo Fisher Scientific, dilution 1:100 in the blocking buffer) and Alexa Fluor 488 goat anti-rabbit IgG antibody (Thermo Fisher Scientific, dilution 1:100 in the blocking buffer) for 2 h at RT. After being washed in PBST, the cells were mounted using VECTASHIELD Mounting Medium with DAPI (Vector Laboratories). The specimens were observed using a fluorescence microscope (model BZ-X700; Keyence, Osaka, Japan).

### Real-time qPCR

Total RNA was prepared using an SV Total RNA Isolation System (Promega Biosciences). First-strand cDNA synthesis was performed with total RNA using a SuperScript III First-Strand Synthesis System for RT-PCR (Thermo Fisher Scientific). Synthesized cDNA was used for the PCR analysis with Power SYBR Green Master Mix (Thermo Fisher Scientific) and primers targeting *CCND1* (F: GATCAAGTGTGACCCGGACTG, R: CCTTGGGGTCCATGTTCTGC), *MYC* (F: CCTGGTGCTCCATGAGGAGAC, R: CAGACTCTGACCTTTTGCCAGG), and *GAPDH* (F: GTCTCCTCTGACTTCAACAGCG, R: ACCACCCTGTTGCTGTAGCCAA). Relative expression levels were calculated using the ΔCt method, normalized against *GAPDH* as an internal control, and analyzed using StepOnePlus software (Thermo Fisher Scientific).

### Cell growth assay

A CellTiter-Glo assay (Promega Biosciences) was used to analyze cell growth. Cells were incubated with CellTiter-Glo detection reagent for 10 min according to the manufacturer’s instructions. Luminescence was observed using a Fluoroskan Ascent FL (Thermo Fisher Scientific). Cell growth was calculated with the control group at 100%.

### Migration assay

Cellular migration was evaluated by a Boyden chamber assay with a transwell membrane filter insert (pore size, 8 mm) in a 24-well plate (BD Biosciences). Cells (1 × 10^5^ cells/insert) were seeded with serum-free DMEM/F12 medium on the top chamber, and the bottom chamber was filled with DMEM/F12 medium containing 10% FBS. After incubation for 24 h, cells that passed through the filter were counted by staining with hematoxylin and eosin. Migrated cells were microscopically imaged and quantified by cell counting in five non-overlapping fields at × 100 magnification.

### Statistical analysis

Before statistical analysis, each experiment was repeated three times. The results are expressed as means ± S.D. Statistical analyses were performed with EZR (Saitama Medical Center, Jichi Medical University, Saitama, Japan), a graphical user interface for R (The R Foundation for Statistical Computing, Vienna, Austria) (Kanda 2013). One-way and two-way ANOVA were used for comparison. If the ANOVA showed a significant difference, Tukey’s test was used as a post hoc test. *p* values of less than 0.05 were considered statistically significant.

## Results

### S100A11 is highly expressed in colorectal *cancer*

**Figure 1.**
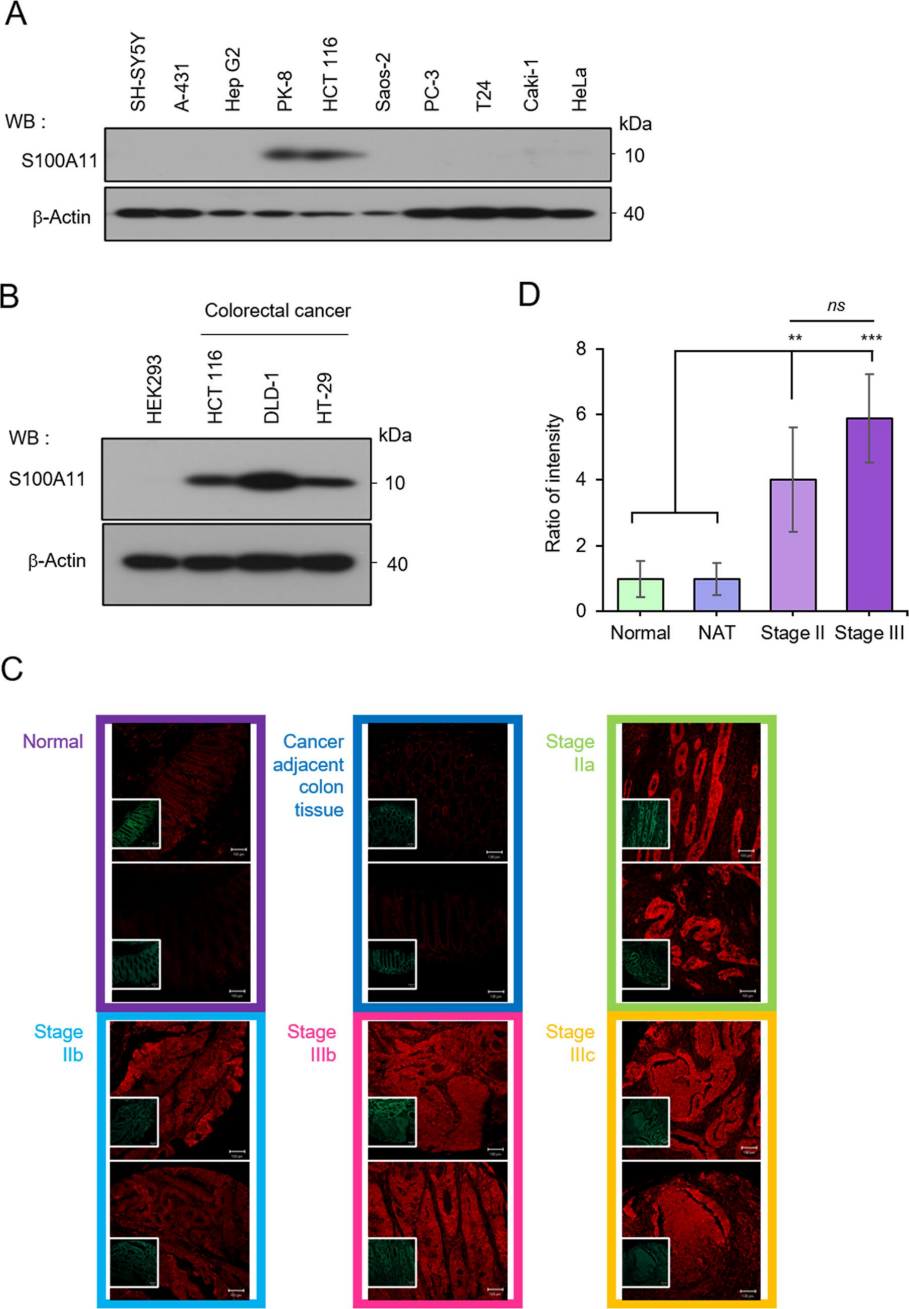
High expression of S100A11 in colorectal cancer. (**A**) Comparison of S100A11 expression in cancer cell lines by Western blot analysis. SH-SY5Y: human neuroblastoma cell line; A-431: human skin epidermoid carcinoma cell line; Hep G2: human hepatocellular carcinoma cell line; PK-8: human pancreas carcinoma cell line; HCT 116: human colon carcinoma cell line; Saos-2: human osteosarcoma cell line; PC-3: human prostate adenocarcinoma cell line; T24: human urinary bladder carcinoma cell line; Caki-1: human kidney carcinoma clear cell line; HeLa: human cervix adenocarcinoma cell line. (**B**) Confirmation of S100A11 high expression in colorectal cancer cell lines by Western blot analysis. (**C**) A colorectal cancer tissue array was immunohistochemically stained for S100A11 (red) and nuclear (green). Staining results were observed microscopically and displayed for the representative images. Bars represent 100 μm. (**D**) The quantified data of the intensities of the S100A11 staining images was exhibited. NAT, normal tissue adjacent; Stage II (Stage IIa and Stage IIb), Stage III (Stage IIIb and Stage IIIc); *ns*, not significant; **p < 0.01, ***p < 0.001.

We have reported that S100A11 is highly expressed in various cancer tissues compared to those in normal tissues, whose relation is highly evident and consistent in the colon, small intestine, pancreas, bladder, and thyroid gland cancers (Saho *et al*. 2016). Therefore, screening was performed using multiple cancer cell lines to investigate further which cancer cells’ expression of S100A11 is elevated in agreement with the result of cancer tissue analysis. As a result, high expression of S100A11 was confirmed in human pancreatic cancer–derived cell line PK-8 and human colorectal cancer–derived cell line HCT 116 (Fig. 1*A*). Although the cell extracts prepared from each cell line were loaded onto SDS-PAGE at 5 μg, β-Actin levels were different because different cell types express different amounts of β-Actin (Fig. 1A). We hence decided to focus on the S100A11 function in colorectal cancer since we had already reported the S100A11 function in pancreatic cancer, where it acts to fuel the proliferation of cancer-associated fibroblasts (CAFs), yielding enriched cancer fibrosis, a pancreatic cancer trait (Takamatsu *et al*. 2019). We confirmed that S100A11 was highly expressed in other same-leagues of cancer cells, DLD-1 and HT-29, and that S100A11 was not expressed in HEK293, a non-colorectal cancer cell line (Fig. 1*B*). The high expression of S100A11 in colorectal cancer cells was further confirmed by immunohistochemistry (Fig. 1*C*). The levels of S100A11 expression tended to be clearly higher in all colorectal cancer tissues compared to normal tissues (Fig. 1*D*).

### S100A11 augments cell growth and cell migration of colorectal *cancer* cells

**Figure 2.**
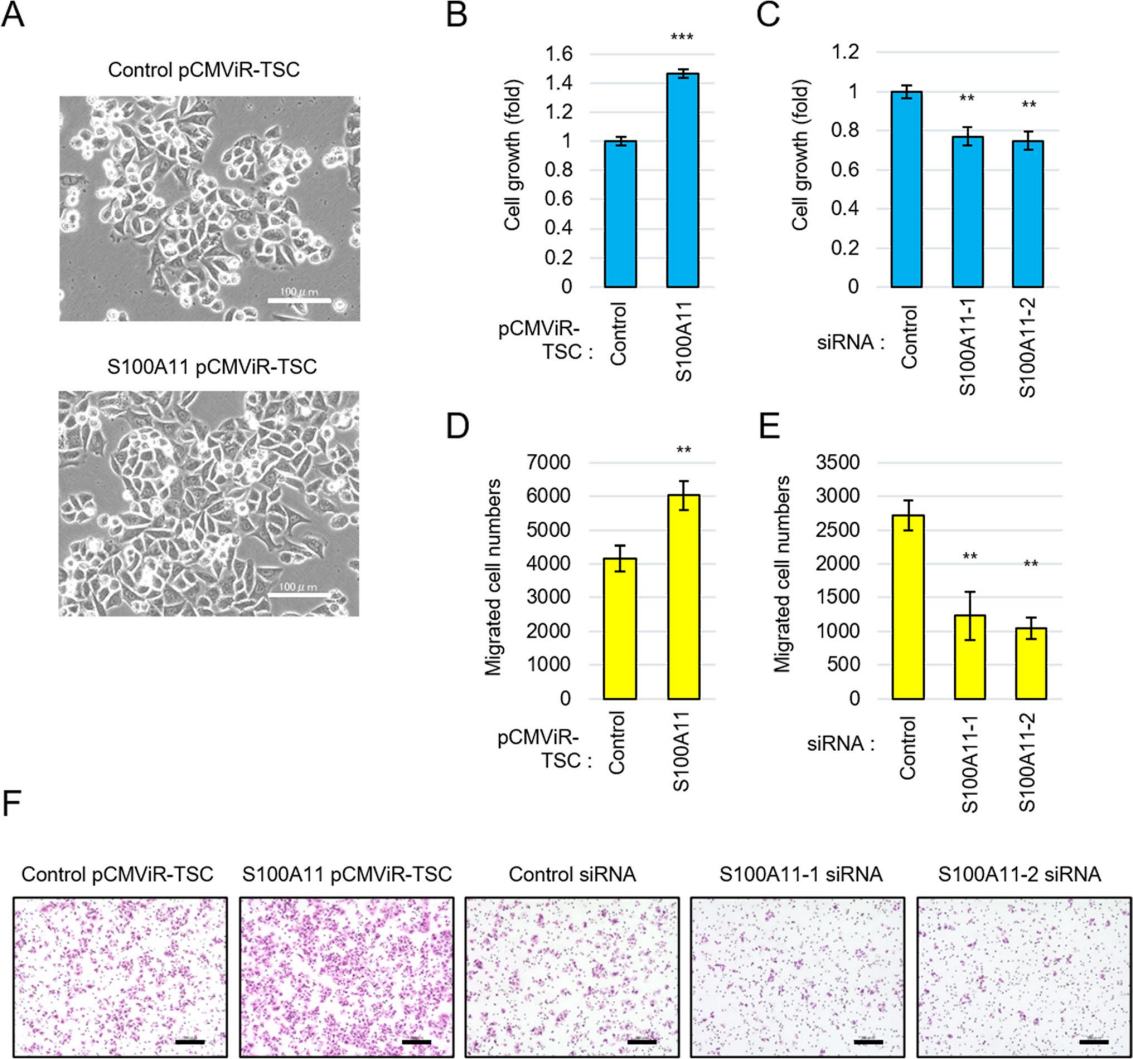
S100A11 is involved in promoting cell proliferation and migration of colorectal cancer cells. DLD-1 cells were overexpressed S100A11 using pCMViR-TSC or knocked down S100A11 using siRNA. (**A**) Cell morphologies of DLD-1 with or without overexpression of S100A11. Scale bar, 100 μm. (**B**, **C**) A CellTiter-Glo assay of the proliferative ability of cells in the indicated conditions. (**D**, **E**) A transwell migration assay of the migration ability of cells in the indicated conditions. (**F**) Representative images of migrated cells. *Scale bar*, 200 μm. **p < 0.01, ***p < 0.001.

**Figure 3.**
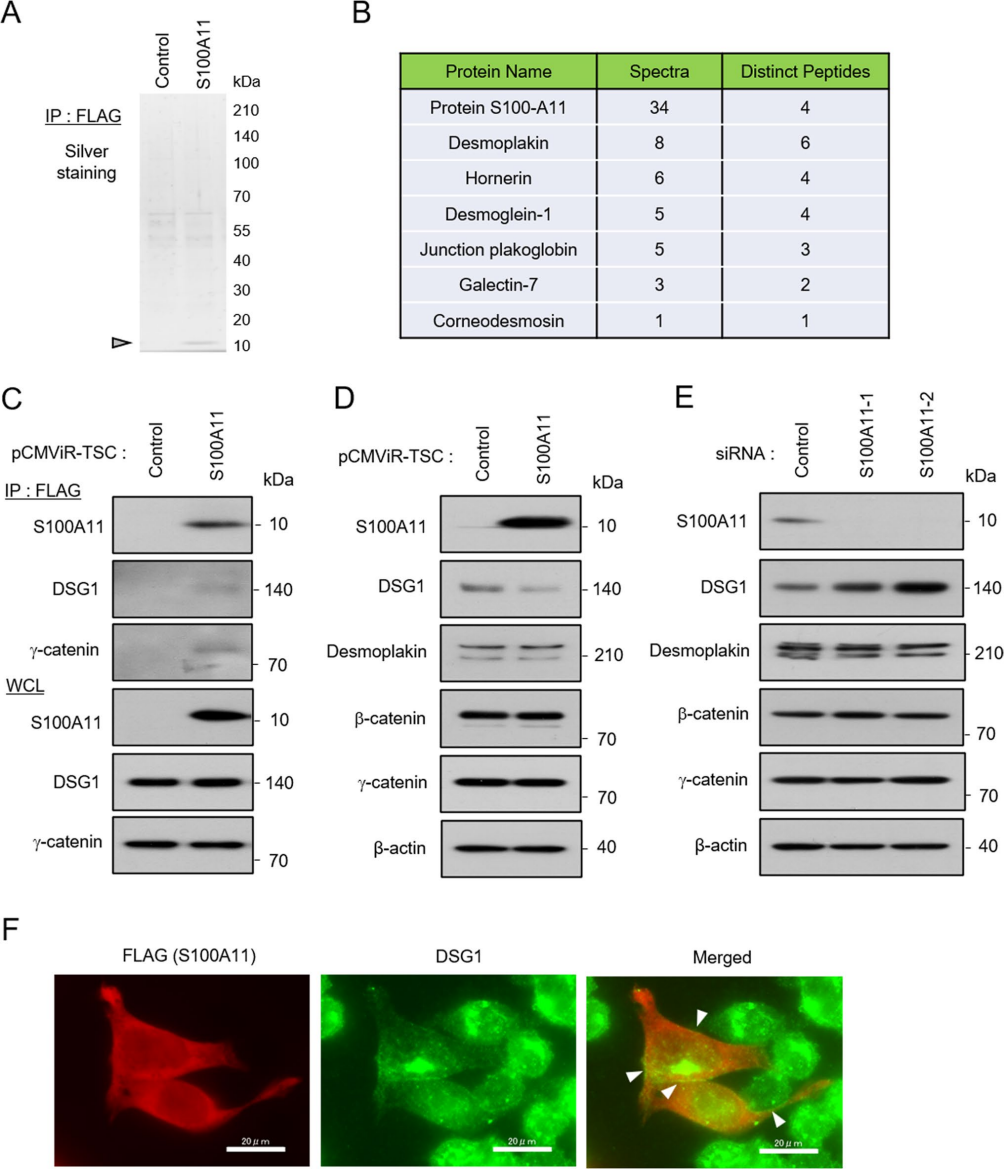
S100A11 binds to desmosome proteins and changes protein levels of DSG1. (**A**, **B**) DLD-1 cells were transfected with control pCMViR-TSC or FLAG-S100A11 pCMViR-TSC for 48 h. These cell lysates were immunoprecipitated with anti-FLAG M2 affinity gel and the immunoprecipitated proteins were eluted. (**A**) Silver staining of immuneprecipitates of S100A11. (**B**) A list of S100A11-interacting proteins detected by LC–MS/MS analysis. (**C**) Confirmation of binding of S100A11 to desmosome proteins by Western blot analysis. (**D**) Overexpression of S100A11 decreases protein levels of DSG1. (**E**) Downregulation of S100A11 increases protein levels of DSG1. (**F**) Subcellular localization of overexpressed S100A11 and DSG1. The cells were immunostained by a FLAG antibody and a DSG1 antibody. *Arrowheads* show the colocalization area of these proteins. *Scale bar*, 20 mm.

To elucidate the role of S100A11 in colorectal cancer, we overexpressed and downregulated the expression of S100A11 in DLD-1 cells and analyzed the phenotypic changes in the cells. Overexpression of S100A11 changed the morphology of DLD-1 cells to become more spindle-shaped (Fig. 2*A*). Overexpression of S100A11 also significantly increased cell proliferation and migration (Fig. 2*B* and *D*). Cell migratory activity was analyzed using a Boyden chamber and HE staining, and an apparent increase in migrated cells was observed (Fig. 2*F*). Inversely, the intrinsic S100A11 expression was lowered using two types of siRNA against S100A11, in which each treated-S100A11 band turned almost invisible (Fig. 3*E*). Under these conditions, we confirmed that the silencing of S100A11 in expression leads suppression of both the proliferation and migration of DLD-1 cells (Fig. 2*C*, *E*, *F*). These results indicate that the fluctuation of S100A11 in expression levels intensively affects growth and migration phenotypes in colorectal cancer cells.

### S100A11 binds to desmosome proteins and alters DSG1 protein levels

To clarify the mechanism by which S100A11 regulates proliferation and migration in colorectal cancer cells, we tried to identify S100A11-binding proteins by immunoprecipitation of the overexpressed S100A11 since the forced expression of it highly contributed to the above phenotypes’ manifestation. Silver staining of the immunoprecipitates of the overexpressed foreign S100A11 allowed us to visualize expressed S100A11 (pointed by arrowhead) and other proteins as bands (Fig. 3*A*), allowing us to proceed to the next protein identification step. LC–MS/MS analysis of the direct trypsinized-immunoprecipitates skipping a series of procedures, loading to SDS-PAGE, gel-silver staining, and cutting the pieces of the bands, enabled us to detect the proteins comprehensively, whose readout list is shown in Fig. 3*B*. The candidate S100A11 binding proteins natured many desmosome proteins such as desmoplakin, desmoglein-1 (DSG1), junction plakoglobin (γ-catenin), and corneodesmosin. Parts of the interactions (S100A11/DSG1 and γ-catenin) that we had an interest in were confirmed by western blotting of immunoprecipitates (Fig. 3*C*). To study the significance of S100A11 interaction with the desmosome proteins, we compelled up or down the expression of S100A11 by the method similar to that described in Fig. 2 and analyzed the levels of the identified proteins. Overexpression of S100A11 decreased DSG1 protein levels, while desmoplakin, β-catenin, and γ-catenin remained unchanged (Fig. 3*D*). Conversely, downregulation of S100A11 increased DSG1 protein levels (Fig. 3*E*). Partial colocalization of the overexpressed S100A11 and DSG1 was observed at the cell edges, and DSG1 protein levels were decreased in the cells overexpressing S100A11 compared to those in the non-overexpressing cells (Fig. 3*F*). In concurrent with the findings, the S100A11 overexpressing cells shape-changed to a more spindle-like morphology compared to those of non-overexpressing cells (Fig. 3*F*). These results suggest that S100A11 interacts with desmosome proteins and affects growth and migration by regulating DSG1 protein levels.

### S100A11 activates the TCF signaling pathway to increase expression of downstream genes

**Figure 4.**
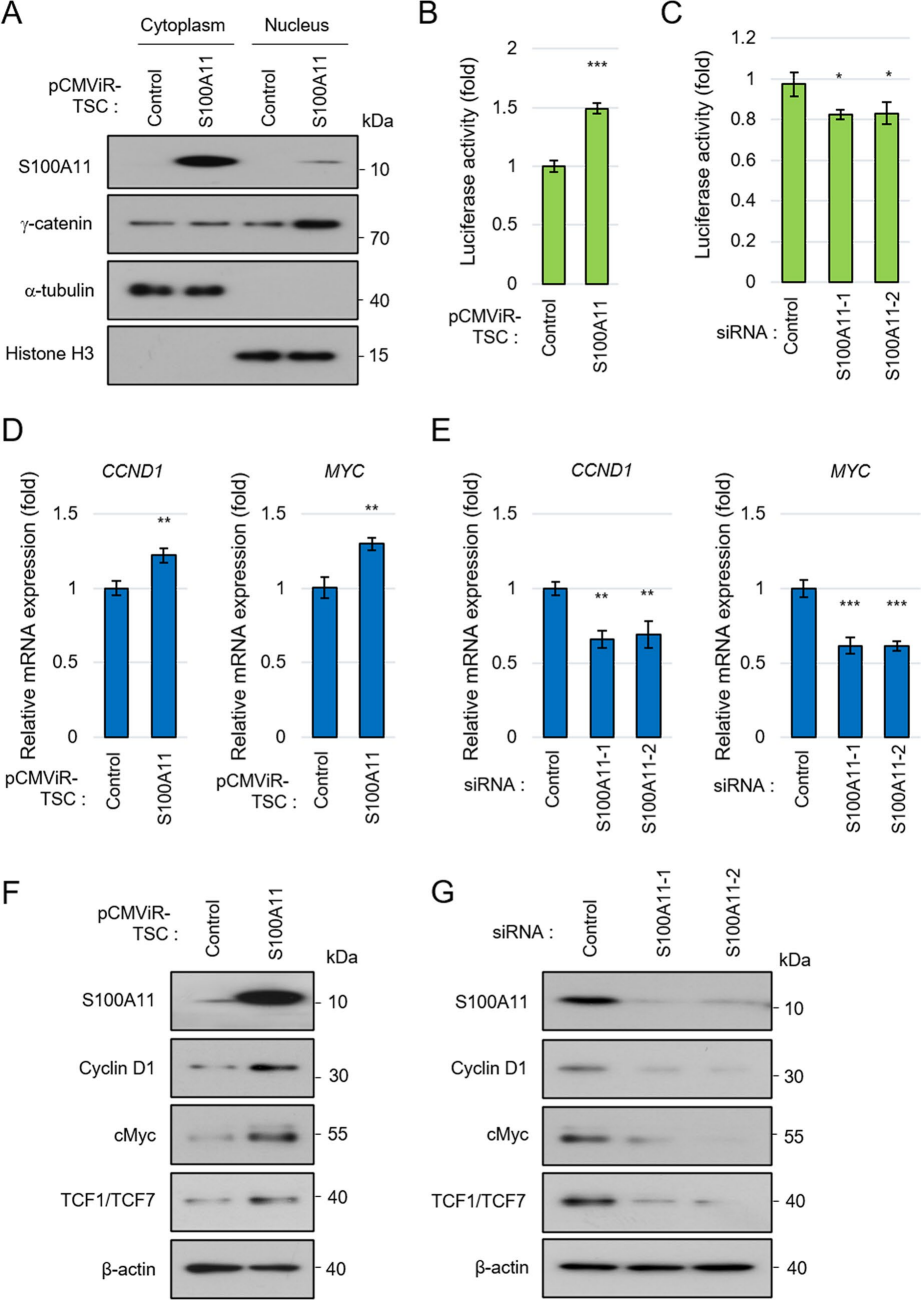
S100A11 activates TCF signaling pathway. (**A**) Overexpression of S100A11 increases the nuclear translocation of γ-catenin. The cells were fractionated to the cytoplasm and nuclear fraction. (**B**, **C**) Luciferase reporter assay using TCF promoter. DLD-1 cells were transfected with pGL4.49, GFP pCMViR-TSC, and S100A11 pCMViR-TSC (**B**) or S100A11 siRNA (**C**). (**D**, **E**) qRT-PCR analysis of TCF target genes (*CCND1* and *MYC*) under overexpression of S100A11 (**D**) or downregulation of S100A11 (**E**). (**F**, **G**) Western blot analysis of TCF-target-genes expression (Cyclin D1, cMyc, and TCF1/TCF7) under overexpression of S100A11 (**F**) or downregulation of S100A11 (**G**). *p < 0.05, **p < 0.01, ***p < 0.001.

Since S100A11 levels highly affect the protein level of DSG1, we hypothesized that S100A11 might regulate the dissociation of γ-catenin from the desmosome through DSG1. A desmosome is a typical molecular group of plasma membrane that plays a crucial role in cell–cell adhesion and signal transduction, and γ-catenin is part of the desmosome (Lessey *et al*. 2022). When dissociated from the desmosome, γ-catenin translocates into the nucleus, regulating the expression of TCF target genes together with TCF (Maeda *et al*. 2004). It has also been reported that γ-catenin promotes nuclear translocation of β-catenin, which is involved in the development of acute myeloid leukemia (Morgan *et al*. 2013). To confirm the subcellular localization of γ-catenin, cells were fractionated into two fractions, cytoplasm and nuclear parts, under the overexpression condition of S100A11. The resulting two fractionation parts were confirmed by each fraction marker, α-Tubulin and Histone H3, for their proper separation of the desired quality (Fig. 4*A*). As expected, overexpression of S100A11 increased the nuclear translocation of γ-catenin, and a part of S100A11 also localized in the nucleus (Fig. 4*A*). Overexpression of S100A11 also increased the activity of the TCF promoter-containing luciferase reporter (Fig. 4*B*), and knockdown of S100A11 inversely decreased it (Fig. 4*C*). The mRNA expression of *CCND1* and *MYC*, which are TCF target genes, also changed depending on the expression level of S100A11 (Fig. 4*D*, *E*). Furthermore, at the protein level, overexpression of S100A11 enhanced the expression of cyclin D1, c-Myc, and TCF1/TCF7 (Fig. 4*F*). TCF1/TCF7 itself is also one of the TCF target genes (Zhu *et al*. 2015). Downregulation of S100A11 significantly decreased their expression (Fig. 4*G*). These results suggest that S100A11 activates the TCF signaling pathway by the nuclear translocation of γ-catenin and regulating TCF1/TCF7 expression.

### S100A11 enhances colorectal *cancer* proliferation and migration via the TCF signaling pathway

**Figure 5.**
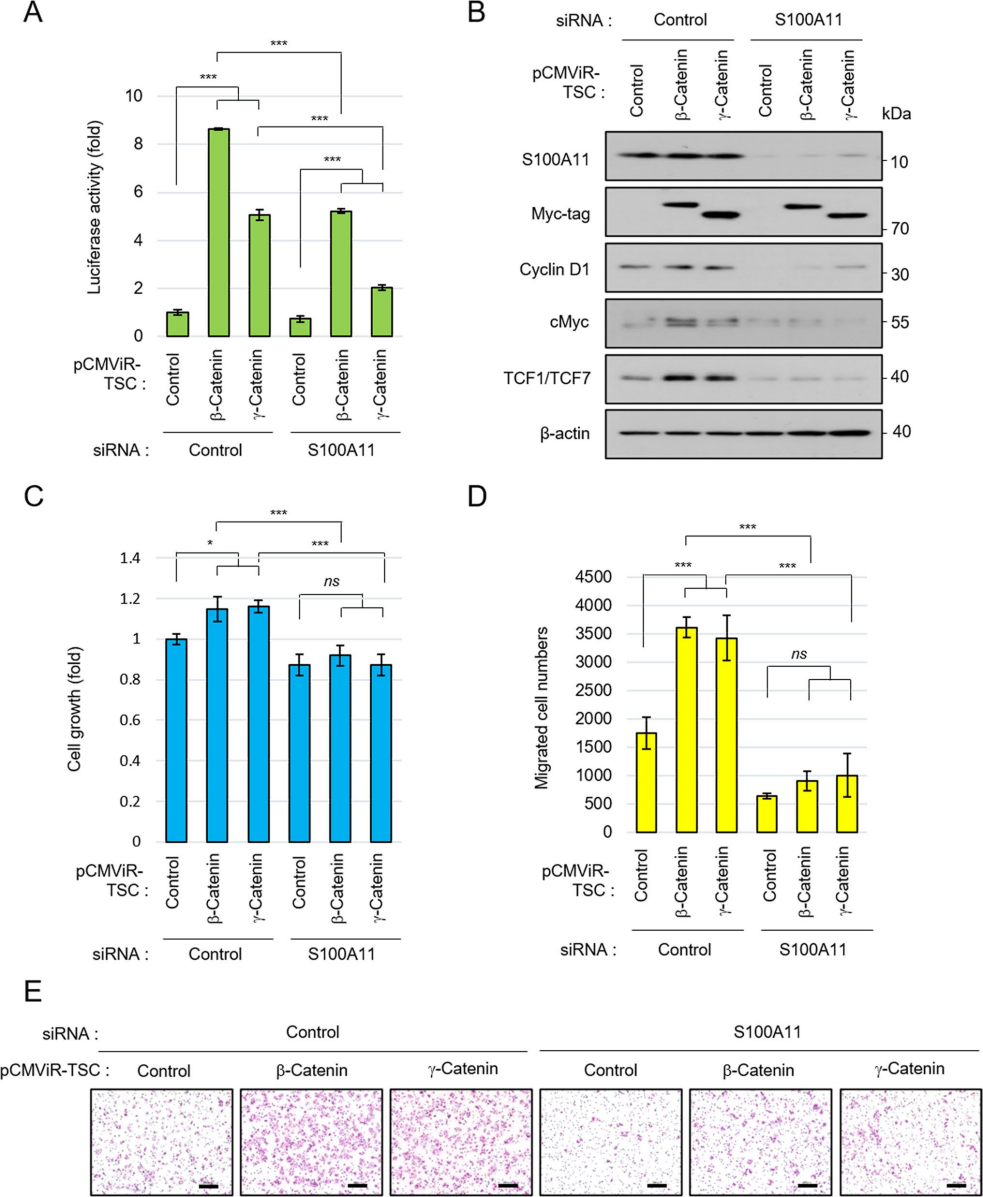
S100A11 augments proliferation and migration of colorectal cancer cells through catenin-TCF signaling pathway. DLD-1 cells were transfected with Control siRNA or S100A11 siRNA for 24 h, and then were transfected with Control pCMViR-TSC, β-catenin pCMViR-TSC, or γ-catenin pCMViR-TSC for another 24 h. (**A**) Luciferase reporter assay using TCF promoter. In this experiment, pGL4.49 and GFP pCMViR-TSC were co-transfected with catenin pCMViR-TSC. (**B**) Knockdown of S100A11 reduces TCF target gene expression induced by overexpression of catenin. (**C**) A CellTiter-Glo assay of the proliferative ability of cells in the indicated conditions. (**D**) A transwell migration assay of the migration ability of cells in the indicated conditions. (**E**) Representative images of migrated cells. *Scale bar*, 200 μm. *ns*, not significant, *p < 0.05, ***p < 0.001.

Next, we investigated whether S100A11 is involved in colorectal cancer progression by activating the TCF signaling pathway. To investigate it, we overexpressed β-catenin and γ-catenin under the suppression of S100A11 expression and analyzed changes in the TCF signaling pathway, proliferation, and migration of DLD-1 cells. Overexpression of β-catenin and γ-catenin increased the TCF promoter activity. Still, suppression of S100A11 expression reduced the activity (Fig. 5*A*). Expression of TCF target genes was also increased by overexpression of catenins but decreased by knockdown of S100A11 expression (Fig. 5*B*). Correlating with changes in these TCF target genes, colorectal cancer proliferation and migration were also enhanced by overexpression of catenins, but decreased by suppression of S100A11 expression (Fig. 5*C*–*E*). These results indicated that S100A11 regulates colorectal cancer growth and migration by activating the TCF signaling pathway.

## Discussion

In this study, we revealed that S100A11 is elevated in colorectal cancer cells, and the increased level is actively conducive to promoting proliferative and migrative outgrowth. Our efforts succeeded in identifying new S100A11 binding partners, desmosome proteins, which became a clue to uncover an essential part of S100A11 role within colorectal cancer cells; i.e., the desmosomal interaction of S100A11 regulates the expression of the desmosome component DSG1, activates the catenin-TCF signaling pathway via cytoplasmic release of γ-catenin, and is involved in the growth and migration of colorectal cancer.

Researchers studying S100 proteins have compiled a growing mass of evidence that changes in the expression level of the S100 proteins affect cancer growth and metastasis. For example, it has been reported that S100A4 is upregulated in breast cancer and is involved in promoting cancer metastasis (Jenkinson *et al*. 2004; Wang *et al*. 2012). S100P is highly expressed in pancreatic cancer and is reported to be involved in proliferation and metastasis (Arumugam *et al*. 2005; Barry *et al*. 2013). Therefore, they are expected to be beneficial as a potent diagnostic marker for breast and pancreatic cancer. Besides S100A4 and S100P, we confirmed that the expression of S100A11 is enhanced in colorectal cancer, so the high expression of S100A11 in colorectal cancer leads us to a prediction of the cancer progression state. Although the increased expression of S100A11 may benefit clinical usage, the question of how increased expression of S100A11 is induced in cancer cells remains elusive. One cue may come from our previous studies, where we reported that S100A11 significantly increased its expression when fibroblast cells achieved a high cell density, termed a confluent condition (Sakaguchi *et al*. 2000). Conceivably, confluent-mediated fastening stress may trigger the induction of high S100A11 levels in cells, whose event may match with a hypoxia condition of the overgrowing big-sized tumor with poor nutrition, which condition leverages cancer cells to run away from the stressed condition, i.e., resulting in invasive metastasis. S100A11 overexpression may be involved in part of the cancer’s aggressive shifting in such a stressed tumor context. Owing to the relatively high expression nature of S100A11 in colorectal cancer cells, even in sparsely occupied conditions in culture, the machinery of S100A11 expression regulation in cancer cells at molecular levels is our next subject to clarify.

Our next question is how overexpression of S100A11 gives benefits such as active proliferative and migrative abilities to colorectal cancer cells. Owing to the presence of many binding partners of S100A11 despite its small body, S100A11 is localized both intracellularly and extracellularly, and thereby, it has multiple cellular functions that orthogonally contribute to the same function such as proliferation and migration in some cases (Sakaguchi and Huh 2011). Take normal human fibroblasts, for instance; the distribution of S100A11 is mainly cytoplasm via binding with filamentous actin polymers in part in growing semiconfluent condition, and when the cells enter into confluent state, S100A11 dissociates from actin fibers and moves to and accumulates in the nuclei by the binding with nucleolin, resulting in growth suppression, which is involved in contact inhibition of normal cells (Sakaguchi *et al*. 2003). Interestingly, the nuclear movement mechanism is ill at molecular levels in cancer cells that fail to be contact inhibition. Cancer cells, instead, use S100A11 for their growth advantage through the secretion of S100A11 into the outside fluid, which stimulates receptors for advanced glycation end product (RAGE) on cancer cells in an autocrine manner (Sakaguchi *et al*. 2008). For the extracellularly releasing process, S100A11 is required to meet PEX14, a peroxisome membrane protein, leading to peroxisomal co-option, subsequent homodimerization, and secretion in cancer cells (Saho *et al*. 2016). Cancer cells are better at using S100A11 for other migration. Jaiswal *et al*. reported that S100A11 is required for plasma membrane repairment upon binding with annexin A2 on the inner membrane side, which is beneficial to maintain the invasive migration activity in breast cancer cells since the cell membrane has trauma in part through the movement process (Jaiswal *et al*. 2014).

Besides those as described above, we also found a vital role of S100A11 localized on the inner membrane with the complex of desmosome proteins, where S100A11 regulates the expression level of DSG1, a component protein of desmosome, by which S100A11 activates the TCF pathway via promoting nuclear translocation of γ-catenin from the desmosome, eventually leading to upregulation of colorectal cancer aggressiveness caused by proliferation and migration advantages. Hence, we considered that S100A11 coordinately and orthogonally functions to proliferation or migration through the pleiotropic routes with annexin A1 (Sakaguchi *et al*. 2007) and desmosome proteins on the inner plasma membrane and with RAGE on the outside plasma membrane. Therefore, how many parts of the newly identified S100A11-mediated TCF pathway are involved in colorectal cancer proliferation and migration compared to another path is required to dissect, whose theme also contains solving how S100A11 regulates the protein level of DSG1 so that those critical issues are our future task.

## Supplementary Information

Below is the link to the electronic supplementary material.

Supplementary file1 (DOCX 379 KB)
