# Correction: S100A11 is involved in the progression of colorectal cancer through the desmosome-catenin-TCF signaling pathway

**DOI:** 10.1007/s11626-024-00940-0

**Published:** 2024-06-27

**Authors:** Jin Zhou, Hitoshi Murata, Nahoko Tomonobu, Naoko Mizuta, Atsuko Yamakawa, Ken-ichi Yamamoto, Rie Kinoshita, Masakiyo Sakaguchi

**Affiliations:** 1 Department of Cell Biology, Okayama University Graduate School of Medicine, Dentistry and Pharmaceutical Sciences, 2-5-1 Shikata-Cho, Kita-Ku, Okayama, 700-8558 Japan; 2 Medical Oncology Department of Gastrointestinal Tumors, Liaoning Cancer Hospital & Institute, Cancer Hospital of the Dalian University of Technology, Shenyang, Liaoning China

**Correction: In Vitro Cellular & Developmental Biology - Animal**

10.1007/s11626-024-00930-2

The given name of coauthor Naoko Mizuta was misspelled (as "Nahoko") in the article as originally published.

The original article has been corrected.

