# Supplementary material for: S100A11 is involved in the progression of colorectal cancer through the desmosome-catenin-TCF signaling pathway
Source: In Vitro Cell Dev Biol Anim. 2024 Jun 6;60(10):1138–49. doi: 10.1007/s11626-024-00930-2 (PMC11655616; doi:10.1007/s11626-024-00930-2)
Supplement: Supplementary file 1 — Supplementary file1 (DOCX 379 KB) [file 11626_2024_930_MOESM1_ESM.docx]

**Supporting Information for**

**S100A11 is involved in the progression of colorectal cancer through the desmosome-catenin-TCF signaling pathway**

Jin Zhou, Hitoshi Murata, Nahoko Tomonobu, Nahoko Mizuta, Atsuko Yamakawa, Ken-ichi Yamamoto, Rie Kinoshita, Masakiyo Sakaguchi

Correspondence should be addressed to Dr. Hitoshi Murata

Mailing address: Department of Cell Biology, Okayama University Graduate School of Medicine, Dentistry and Pharmaceutical Sciences, 2-5-1 Shikata-cho, Kita-ku, Okayama 700-8558, Japan, Tel: +81-86-235-7397, Fax: +81-86-235-7400, E-mail: murata@md.okayama-u.ac.jp

**This file includes:**

Fig. S1

**
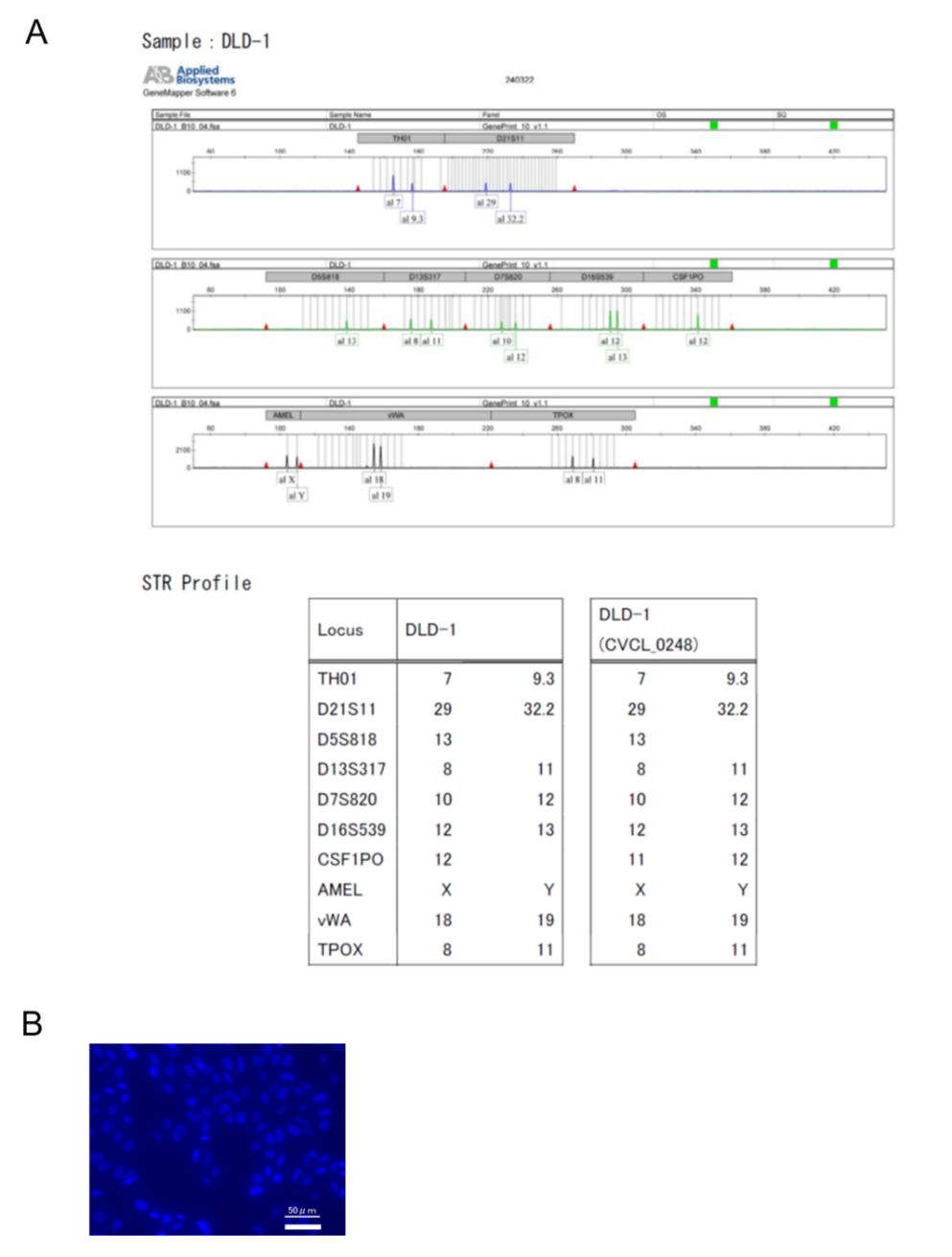
**

**Figure S1.** Cell line authentication of DLD-1 (A) The DLD-1 cell line was authenticated by the STR analysis. The STR profiles of DLD-1 were not completely matched with those of DLD-1 (CVCL_0248). However, the evaluation value (EV) between DLD-1 and DLD-1 (CVCL_0248) was 0.97, which was high enough that the STR profiles of DLD-1 were the same as those of DLD-1 (CVCL_0248). Therefore, two cell lines were considered to be the identical cell strains. (B) Hoechst staining of DLD-1 cells. Scale bar: 50 μm.
